# Reflections on the Inclusion of Direct-Care Physicians as Educators in Community Hospitals

**DOI:** 10.1590/0100-6991e-20243856-en

**Published:** 2024-12-02

**Authors:** RENÉ SCALET DOS SANTOS, DANIELLE BRUGINSKI, ANA FÁTIMA VOLKMANN, JOSÉ ANDRADE MOURA

**Affiliations:** 1 - Faculdades Pequeno Príncipe, Faculdade de Medicina - Curitiba - PR - Brasil; 2 - Escola Bahiana de Medicina e Saúde Pública, Clínica Médica - Salvador - BA - Brasil

**Keywords:** Education, Medical, Preceptorship, Hospitalsm, Community, Unified Health System, Schools, Medical, Educação Médica, Preceptoria, Hospitais Comunitários, Sistema Único de Saúde, Faculdades de Medicina

## Abstract

This paper discusses the increasing trend of direct-care physicians taking on teaching roles in community hospitals, both in the United States and Brazil. It highlights the challenges faced by these physicians, who often lack formal pedagogical training and dedicated time for teaching. The text emphasizes the need for structured support, faculty development programs, and collaboration with academic centers to ensure the quality of education in these settings. It also underscores the potential benefits of this model, such as increased access to medical training in underserved areas and a more hands-on learning experience for students. Overall, the document calls for a thoughtful and comprehensive approach to integrating direct-care physicians as educators, ensuring that this practice benefits both the physicians themselves and the quality of medical education.

## RESUMO

Este artigo discute os desafios e oportunidades da inserção de médicos assistenciais como educadores em hospitais comunitários, tanto nos EUA quanto no Brasil. A expansão das escolas médicas e a escassez de médicos levaram à nomeação de médicos assistenciais para funções de ensino, muitas vezes sem treinamento formal. Médicos nos EUA e no Brasil enfrentam desafios semelhantes, como falta de tempo, preparação e apoio institucional para o ensino. Hospitais comunitários oferecem oportunidades únicas de aprendizado, mas podem ter recursos limitados. O artigo destaca a necessidade de programas de capacitação docente, materiais didáticos e colaboração com centros acadêmicos para apoiar esses médicos. Conclui-se que, com o apoio adequado, a inserção de médicos assistenciais como educadores pode beneficiar tanto a formação médica quanto os serviços de saúde em áreas carentes.

## INTRODUCTION

The article “The Accidental Teacher-Direct-Care Physicians Increasingly Placed in Teaching Roles”, by Sweigart et al., addresses how primary care physicians are being increasingly assigned to teaching roles in community hospitals in the United States, often without adequate formal preparation. This trend is also observed in Brazil, where the expansion of medical schools and residency programs leads clinical physicians to assume preceptorship roles without academic experience^1^ ^,^ ^2^. From the 2010s onwards, with the More Doctors Program, there was an unbridled expansion of medical courses in Brazil, resulting in an increase in schools and vacancies, particularly in private institutions^3^. This policy has accelerated the training of new doctors, many of whom without experience or pedagogical preparation, enter teaching functions directly or assuming clinical positions in regions with a shortage of professionals. In addition, the lack of specific training and the unequal scenario of the offer of medical residency make the phenomenon of the “accidental professor” even more evident^4^.

### Bioethical Principles in Community Medical Education

Medical education in community settings should be guided by the principles of bioethics described by Beauchamp and Childress: autonomy, beneficence, non-maleficence, and justice^5^. Respecting autonomy means allowing students to express their educational needs and actively participate in the learning process. Beneficence and non-maleficence require preceptors to promote students’ educational growth while minimizing the harms associated with the emotional overload of a challenging work environment. Finally, justice entails ensuring equitable access to learning opportunities while respecting differences among students.

### Critical Analysis of the North American Context

In the United States, the transformation of community hospitals into teaching centers responds to the growing demand for medical training in less centralized areas. Sweigart et al. point out that, although the combination of practice and teaching may seem like a natural extension of the medical role, the lack of pedagogical training and protected time for educational activities compromises professionals’ quality of teaching and well-being^1^. Preceptor development programs, with online modules and role-plays, improve teaching competence and reduce the challenges of these roles in community settings^6^.

### Comparison with the Brazilian Reality

The expansion of medical education in Brazil reflects the need to provide health education in areas with lower coverage. Since the implementation of the More Doctors Program, there has been a significant increase in medical schools and undergraduate vacancies, but the creation of residency vacancies has not followed suit^3^. This has resulted in newly graduated doctors taking on teaching roles, often without pedagogical training. This practice questions the completeness of medical training in Brazil, as medical residency is not mandatory for clinical or educational practice as it is in other countries^4^.

In addition, the teaching scenario in the Public Health System (SUS) faces unique challenges. Community hospitals often lack educational infrastructure and institutional support, which impairs students’ experience and increases the burden on preceptors^2^. The lack of financial incentives and dedicated time make it even more difficult to consolidate quality education in these environments^7^.

### Benefits and Limitations of Teaching in Community Settings

Although community environments have structural limitations, such as less case complexity and scarcity of pedagogical resources, they offer unique learning opportunities. Students have direct access to procedures and patients in places with less competition, favoring a more personalized and practical learning experience^1^. However, the effectiveness of teaching in these environments depends on the integration between theoretical knowledge and practical supervision, something often hampered by instructors’ lack of preparation^2^ ^,^ ^8^.

### Proposed Solutions and Future Paths

In the face of these challenges, it is critical to implement faculty development programs. Professor training initiatives should include workshops, structured feedback, and simulations, in addition to addressing specific competencies for community preceptors^6^ ^,^ ^9^. The integration of reliable professional activities (RPAs) into the medical curriculum can improve the assessment of educational progress and ensure that the necessary competencies are achieved^5^.

The adoption of teaching-service integration models, such as COAPES, can also strengthen the alignment between medical education and the SUS demands. In addition, partnerships with academic centers can provide technical and pedagogical support to community preceptors, including telementoring programs^10^.

Finally, institutional incentives, such as credits in continuing medical education and additional remuneration, can increase the engagement of preceptors and improve the quality of teaching in community hospitals^7^.

## CONCLUSION

The reality of clinical physicians as instructors in community hospitals is challenging, but with the potential to enrich medical education and strengthen health services in underserved areas. To realize these benefits, it is necessary to invest in teacher development programs, integrate bioethical principles into teaching, and strengthen partnerships between academic and community institutions. These actions can transform the role of the community preceptor, ensuring that it is not only an additional responsibility, but an opportunity for educational growth and innovation in medical education^3^ ^,^ ^4^.
